# The prevalence and predictive value of dipstick urine protein in HIV-positive persons in Europe

**DOI:** 10.7448/IAS.17.4.19561

**Published:** 2014-11-02

**Authors:** Amanda Mocroft, Lene Ryom, Giuseppe Lapadula, Peter Reiss, Anders Blaxhult, Hansjakob Furrer, Galyna Kutsyna, Jose Gatell, Josep Begovac, Ole Kirk, Jens Lundgren, for EuroSIDA in EuroCoord

**Affiliations:** 1 Department of Infection and Population Health, University College London, London, UK; 2 Copenhagen HIV Programme, Department of Infectious Diseases, University of Copenhagen/Rigshospitalet, Copenhagen, Denmark; 3 Clinica di Malattie Infettive, “San Gerardo” Hospital, Monza, Italy; 4 Division of Infectious Diseases/Department of Global Health, University of Amsterdam, Academic Medical Centre, Amsterdam, Netherlands; 5 Department of Infectious Diseases, Venhaelsan-Sodersjukhuset, Stockholm, Sweden; 6 Department of Infectious Diseases, Bern University Hospital, University of Bern, Bern, Switzerland; 7 Regional AIDS Centre, Luhansk State Medical University, Luhansk, Ukraine; 8 Infectious Diseases and AIDS, Hospital Clinic i Provincial, Barcelona, Spain; 9 Department of Infectious Diseases, University Hospital of Infectious Diseases, Zagreb, Croatia

## Abstract

**Introduction:**

Proteinuria (PTU) is an important marker for the development and progression of renal disease, cardiovascular disease and death, but there is limited information about the prevalence and factors associated with confirmed PTU in predominantly white European HIV+ persons, especially in those with an estimated glomerular filtration rate (eGFR) of 60 mL/min/1.73 m^2^.

**Patients and methods:**

Baseline was defined as the first of two consecutive dipstick urine protein (DPU) measurements during prospective follow-up >1/6/2011 (when systematic data collection began). PTU was defined as two consecutive DUP >1+ (>30 mg/dL) >3 months apart; persons with eGFR <60 at either DPU measurement were excluded. Logistic regression investigated factors associated with PTU.

**Results:**

A total of 1,640 persons were included, participants were mainly white (n=1,517, 92.5%), male (n=1296, 79.0%) and men having sex with men (n=809; 49.3%). Median age at baseline was 45 (IQR 37–52 years), and CD4 was 570 (IQR 406–760/mm^3^). The median baseline date was 2/12 (IQR 11/11–6/12), and median eGFR was 99 (IQR 88–109 mL/min/1.73 m^2^). Sixty-nine persons had PTU (4.2%, 95% CI 3.2–4.7%). Persons with diabetes had increased odds of PTU, as were those with a prior non-AIDS [1] or AIDS event and those with prior exposure to indinavir. Among females, those with a normal eGFR (>90) and those with prior abacavir use had lower odds of PTU (Figure 1).

There was no significant association between past or current use of tenofovir, lopinavir, atazanvir (boosted or unboosted) or any other boosted PI and PTU (p>0.2). During 688.2 person-years of follow up (PYFU), three persons developed chronic kidney disease (CKD; confirmed [>3 months apart] eGFR<60); 2/685 (0.3%) without PTU and 1/38 (2.8%) with PTU (p=0.032). The crude incidence of CKD in those with baseline PTU and eGFR>60 was almost 10 times higher than in those without baseline PTU and eGFR>60 (rate ratio 9.61; 95% CI 0.87–105.9, p=0.065).

**Conclusions:**

One in 25 persons with eGFR>60 had confirmed proteinuria at baseline. Factors associated with PTU were similar to those associated with CKD. The lack of association with antiretrovirals, particularly tenofovir, may be due to the cross-sectional design of this study, and additional follow-up is required to address progression to PTU in those without PTU at baseline. It may also suggest other markers are needed to capture the deteriorating renal function associated with antiretrovirals may be needed at higher eGFRs. Our findings suggest PTU is an early marker for impaired renal function.

**Figure 1:**
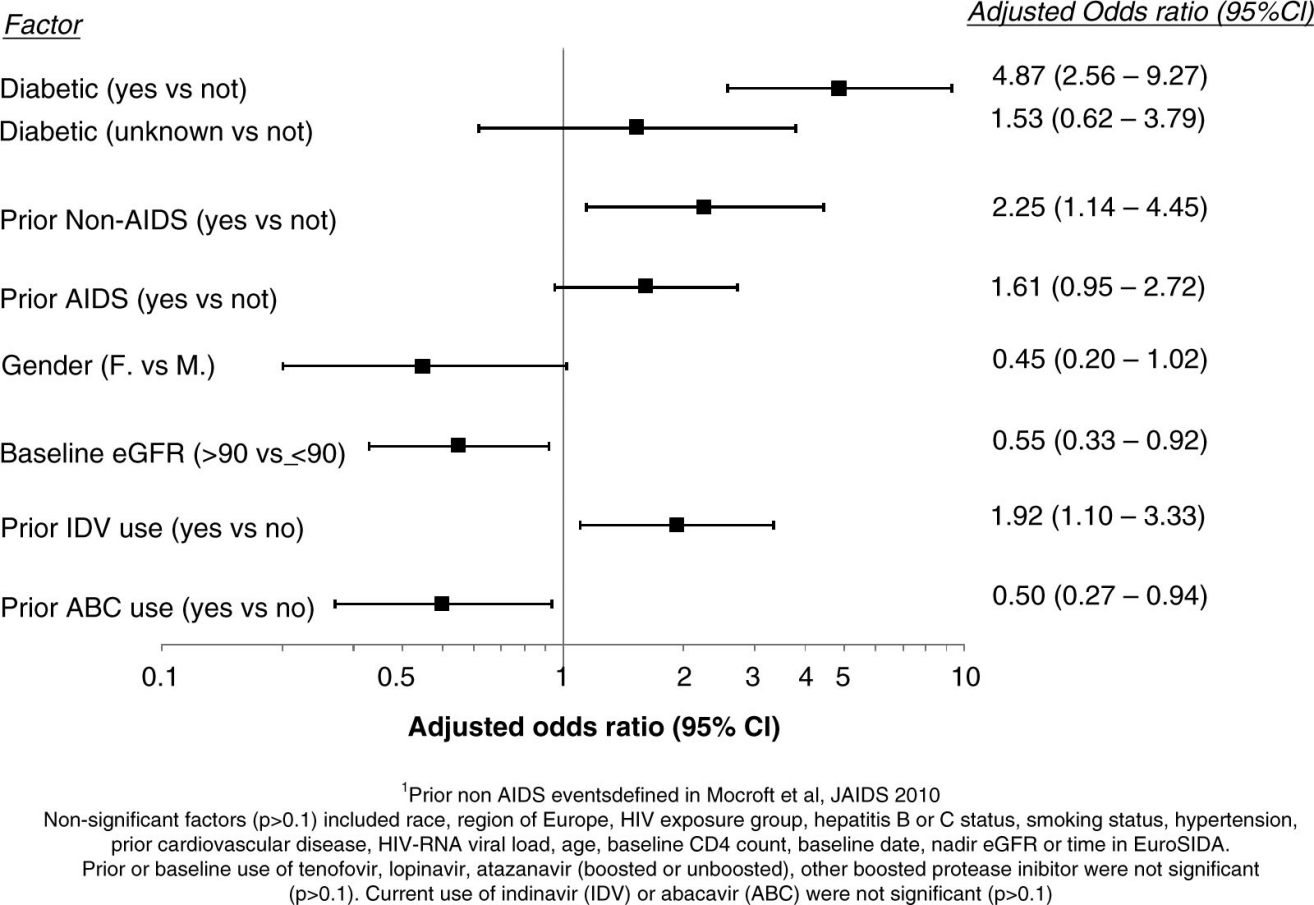
Adjusted odds ratio of baseline PTA in persons with normal (>60) eGFR PTA: 2 consecutive dipstick urine proteinuria≥1+.
